# Application of Microsponge Drug Platform to Enhance Methotrexate Administration in Rheumatoid Arthritis Therapy

**DOI:** 10.3390/pharmaceutics16121593

**Published:** 2024-12-13

**Authors:** Noemi Fiaschini, Patrizia Nadia Hanieh, Daniela Ariaudo, Rita Cimino, Carlo Abbate, Elena Romano, Francesca Cavalieri, Mariano Venanzi, Valeria Palumbo, Manuel Scimeca, Roberta Bernardini, Maurizio Mattei, Alberto Migliore, Antonio Rinaldi

**Affiliations:** 1 Nanofaber S.r.l., Via Anguillarese 301, 00123 Rome, Italy; noemi.fiaschini@nanofaber.com (N.F.); patrizia.hanieh@nanofaber.com (P.N.H.); 2 Department of Chemical Science and Technologies, University of Rome Tor Vergata, Via Della Ricerca Scientifica 1, 00133 Rome, Italy; daniela.ariaudo@alumni.uniroma2.eu (D.A.); rita.cimino@uniroma2.it (R.C.); bbtcrl01@uniroma2.it (C.A.); francesca.cavalieri@uniroma2.it (F.C.); venanzi@uniroma2.it (M.V.); 3 Centre of Advance Microscopy P. Albertano, Department of Biology, University of Rome Tor Vergata, Via della Ricerca Scientifica Snc, 00133 Rome, Italy; romanoe@uniroma2.it; 4 Department of Experimental Medicine, University of Rome Tor Vergata, 00133 Rome, Italy; valeria.palumbo.25@students.uniroma2.eu (V.P.); manuel.scimeca@uniroma2.it (M.S.); 5 Department of Clinical Sciences and Translational Medicine, University of Rome Tor Vergata, 00133 Rome, Italy; roberta.bernardini@uniroma2.it; 6 Interdepartmental Center for Comparative Medicine, Alternative Techniques and Aquaculture (CIMETA), University of Rome Tor Vergata, Via Montpellier 1, 00133 Rome, Italy; mattei@uniroma2.it; 7 San Pietro Fatebenefratelli Hospital, Via Cassia 600, 00189 Rome, Italy

**Keywords:** microsponge, rheumatoid arthritis, methotrexate, slow delivery system

## Abstract

Background/Objectives: This study aimed to develop a novel nanotechnological slow-release drug delivery platform based on hyaluronic acid Microsponge (MSP) for the subcutaneous administration of methotrexate (MTX) in the treatment of rheumatoid arthritis (RA). RA is a chronic autoimmune disease characterized by joint inflammation and damage, while MTX is a common disease-modifying antirheumatic drug (DMARD), the conventional use of which is limited by adverse effects and the lack of release control. Methods: MSP were synthesized as freeze-dried powder to increase their stability and allow for a facile reconstitution prior to administration and precise MTX dosing. Results: A highly stable and rounded-shaped micrometric MSP, characterized by an open porosity inner structure, achieved both a high MTX loading efficiency and a slow release of MTX after injection. Our drug release assays indeed demonstrated a characteristic drug release profile consisting of a very limited burst release in the first few hours, followed by a slow release of MTX sustained for over a month. By means of a preclinical rat model of RA, the administration of MTX-loaded MSP proved to nearly double the therapeutic efficacy compared to sole MTX, according to a steep reduction in arthritic score compared to control groups. The preclinical study was replicated twice to confirm this improvement in performance and the safety profile of the MSP. Conclusions: This study suggests that the MSP drug delivery platform holds significant potential for clinical use in improving RA therapy by enabling the sustained slow release of MTX, thereby enhancing therapeutic outcomes and minimizing side effects associated with conventional burst-release drug administration.

## 1. Introduction

Rheumatoid arthritis (RA) is an immune-mediated inflammatory disease characterized by hyperplastic synovium, cartilage damage, bone erosion, and different systemic manifestations [1]. It involves the activation of fibroblast-like synoviocytes and monocyte infiltration into the joints, where they differentiate into macrophages [2]. Eventually, this process perpetuates and leads to a condition of chronic illness that severely affects the quality of life of patients. RA affects approximately 0.5% to 1% of the global population, with an onset typically occurring between ages 25 and 60 and an incidence that is rising worldwide [3,4]. Women are three times more likely than men to develop RA, particularly during the postpartum period, and often require therapeutic interventions throughout their lives just to manage symptoms [5,6]. Individuals with RA experience serious symptoms, such as persistent discomfort, swelling, limited joint mobility, and difficulty performing daily activities.

The effective therapy of RA remains an active research area, although several advances have occurred in recent times. For example, a major result was the recognition that early and effective control of inflammation improves the therapeutic outcomes, which emphasized the importance of a personalized treatment and spurred the development of the “treat-to-target” strategy [7]. This approach seeks to adjust the therapy of a given patient based on the level of specific target parameters, helping to achieve better disease control by minimizing joint damage and improving overall quality of life. In addition, advancements in biotechnology and a growing range of treatment options witness the current attention towards RA therapy. Yet, despite all efforts, conventional synthetic DMARDs (disease-modifying antirheumatic drugs) remain the gold-standard first-line therapy for RA today. Clinical trials and observational studies confirm in fact that MTX is the preferred DMARD for RA and juvenile idiopathic arthritis, due to its relatively low cost and favorable efficacy/safety profile [8,9,10]. MTX offers efficacy that is at least equal to, if not better than, other monotherapies [11]. Additionally, MTX has a quicker onset of action and greater treatment durability [12]. Thus, it is relevant to identify better ways to deliver MTX therapy, like in the present study.

Oral MTX administration is still the most common and convenient method for RA treatment, offering significant advantages in terms of patient compliance and ease of self-administration [13,14]. However, oral MTX fails to halt disease progression in 70–80% of patients, as demonstrated in large-scale clinical trials. Furthermore, prolonged and repeated oral administration of MTX is associated with various adverse effects, including gastrointestinal, hepatic, pulmonary, renal, and hematologic complications. Consequently, many patients opt for subcutaneous (SC) injections, recognized as an effective strategy to bypass the gastrointestinal tract [15]. SC administration offers more reliable and efficient treatment regimens, improving drug bioavailability. In particular, MTX administered subcutaneously results in higher blood levels and long-chain MTX-polyglutamate formation, which may exceed the saturation capacity of the reduced folate carrier, a protein critical to drug absorption. Studies such as the CATCH trial demonstrate that SC administration is more effective than oral dosing for RA treatment. This highlights the importance of the administration route over dosage in achieving therapeutic outcomes. However, nausea and vomiting [16,17], caused by elevated peak blood levels of MTX [18,19], along with discomfort and stress associated with SC injections, further reduce patient compliance [20].

While such conventional therapeutic formats remain widely used for their cost-effectiveness, there is a demand for more refined protocols and an increasing interest in micro and nanotechnology systems for MTX delivery [21], such as nanoparticles [22], liposomes [23], dendrimers [24], microspheres [25], and injectable hydrogels [26]. All of them promise to offer improved targeting, effectiveness, and minimal side effects, but all of them still face challenges. For example, injectable hydrogels require frequent administrations (every 14 days), increasing the risk of joint infections, and lack the ability to deliver MTX in a staged manner, such as an initial burst for acute symptoms followed by sustained release for stable disease phases [27]. Microspheres, on the other hand, provide controlled and localized MTX delivery, enhancing therapeutic efficacy while reducing dosing frequency and systemic toxicity [28]; however, their performance relies on polymer properties such as composition, molecular weight, and critical degradation profile. Unmet challenges include potential inflammation from acidic degradation products and high production costs, limiting their widespread use [29]. Instead, nano-delivery platforms exhibit enhanced solubility and targeted delivery of MTX, but their clinical translation is hindered by issues such as cellular toxicity, rapid clearance by the reticuloendothelial system, and extracellular matrix barriers that complicate bioavailability [30]. Many of them, such as liposomes, are challenging to produce and, most notably, are chemically and microbiologically fragile [31]. In addition, most nanoscale systems face regulatory uncertainties as the approval procedures are not well established.

In this context, options entailing the slow delivery of MTX are not available on the market but could offer a way to overcome several of said limitations. Novel delivery systems must be designed to strike a balance between sustained MTX release, safety, and scalability.

In this study, we report the results of the first preclinical application of an advanced drug delivery platform (DDP), called Microsponge (MSP) [32], to administer MTX via subcutaneous injection and enable its sustained release over a time-span of several weeks.

The MSP is a nano-enabled, tunable, biodegradable, and biocompatible polymeric DDP made of microscale near-spherical particles endowed with a nanoscale open porous structure, capable of effectively entrapping active ingredients/drugs and their subsequent controlled release [33,34]. These sponge-like particles can also be regarded as a 3D open network of thin-walled interconnected septa with a very large surface area, while maintaining sufficient mechanical strength to ensure structural stability during drug loading–unloading operations, lyophilization, and gradual biodegradation [35].

While a few prior studies explored the use of similar sponge-like systems for topical and transdermal drug delivery (e.g., [36]), thereby showing the potential benefits of sustained release of drugs in RA, none of them focused on the subcutaneous route.

Our objective in this paper is to demonstrate that the deployment of MSP for the slow release of MTX in RA can radically boost therapeutic efficacy in comparison with pharmaceutical formulations on the market, therefore bettering the quality of life of RA patients by bringing pain relief and minimization of systemic side effects.

The development of a novel MTX therapy mediated by MSP with highly superior performance would be a substantial innovation, capable to displace conventional clinical practice and making it possible, for example, to reduce the frequency of injections or dosage to achieve the same outcome for patients.

To reach this objective, a preclinical proof-of-concept investigation was conducted on a collagen-induced arthritis (CIA) rat model to demonstrate the safety and effectiveness of MSP loaded with MTX for RA treatment. The pathophysiological outcomes were evaluated, and molecular and morphological parameters were compared with conventional subcutaneous drug injections. The animal model study was replicated twice to confirm the findings.

## 2. Materials and Methods

### 2.1. Materials

Di(imidazol-1-yl)methanone (carbonyl di-imidazole, CDI), 2,2′-Diaminodiethyl disulfide dihydrochloride (Cys), [9-(2-carboxy-6-isothiocyanatophenyl)-6-(diethylamino) xanthen-3-ylidene]-diethylazanium-chloride (Rhodamine B isothiocyanate, RBITC), and dipotassium-trisodium-dihydrogen phosphate-hydrogen phosphate-dichloride (phosphate buffered saline, PBS) were obtained from Merck Italia (Milan, Italy). Sodium Hyaluronate (HA, MW 1590 kDa) was purchased from Lifecore Biomedical (Chaska, MN, USA). Commercially available Methotrexate (Reumaflex prefilled syringe, dose 50 mg/mL, Alfasigma S.p.A., Bologna, Italy) was used. All additional reagents and chemicals employed were analytical grade.

### 2.2. Synthesis of the Microsponge

The synthesis of hyaluronic acid-based Microsponge was carried out following a previously reported procedure with some modifications [32,37]. An amount of 20 mg of Cys dissolved in 300 µL of MilliQ water was added to 30 mg of CDI and the cross-linker (CL) was obtained as a precipitate. The mixture was then stirred using a vortex and allowed to incubate for 30 min at room temperature. Afterwards, to solubilize the precipitate, 50 µL of HCl 5M was added until reaching a final pH between 2 and 4. The MSP were obtained by adding 1 mL of a solution of HA 1% in MilliQ water (*w*/*v*) to the cross-linker. After the mix was maintained at 30 °C for 24 h, a white precipitate was obtained, washed three times with MilliQ water, and centrifugated each time for 5 min at 5000 RPM to eliminate unreacted cross-linker and polymer molecules. The precipitate, containing purified MSP, was then frozen for 1 h at −20 °C and placed in a freeze-dryer (Lyovapor L 300, Buchi, Italy) for 48 h, with pressure under 0.1 mbar, and a condenser temperature of −60 °C.

### 2.3. Loading Studies

MTX loading in the platform was achieved using the physical adsorption method [38], where 1 mg of lyophilized MSP was reconstituted with 100 µL of an aqueous solution of MTX 15 mg/mL [39,40]. After, the obtained suspension was incubated in a rotary shaker at room temperature for 24 h. At variable intervals (0, 30 min, 1, 2, 3, and 24 h), the sample was centrifuged at 7000 rpm at room temperature for 5 min [41]. The drug loading efficiency into the MSP was assessed using UV-Visible spectroscopy (Jasco V-550, Jasco Corporation, Tokyo, Japan) to quantify the free MTX present in the supernatant. This method provided an indirect measurement of the amount of drug loaded into the MSP, by analyzing the absorbance peak at 302 nm for MTX and referencing a previously established calibration curve [42].

The percentage of MTX that was successfully incorporated into the MSP compared to the initial amount used, expressed as entrapment efficiency (EE), was estimated according to Equation (1). Alternatively, the loading efficiency (LE) of MSP refers to the payload capacity, which indicates the amount of drug encapsulated per unit weight of the MSP, as calculated by Equation (2) [40]:

(1) $$EE\left(\%\right)=\frac{MTX\;loaded\;amount\;\left(mg\right)}{Initial\;amount\;of\;MTX\;(mg)}\times 100$$

(2) $$LE\left(\%\right)=\frac{MTX\;loaded\;(mg)}{Total\;mass\;of\;MSP\;(mg)}\times 100$$

where the MTX loaded amount was obtained by calculating the difference between the initial amount of added MTX and the amount of free MTX in the supernatant. Once the optimal time for achieving maximum drug entrapment in the MSP was determined, the sample was frozen and placed in a freeze-dryer overnight in dark conditions [43]. This process was carried out to obtain a dosed powder ready to be resuspended immediately before administration in animal studies.

### 2.4. Endotoxin Detection

Since the formulations in this work could eventually be applied to subcutaneous (parenteral) administration in a clinical setting, it is essential that the MSP are sterile and endotoxin-free. This was already accounted for in this study, ensuring that all synthesis procedures were conducted under laminar air flow conditions, using sterilized solvents and materials to prevent microbial contamination. After synthesis, the samples were analyzed to detect the presence of endotoxins by means of ToxinSensor™ Chromogenic LAL Endotoxin Assay Kit by Genescript (Piscataway, NJ, USA). The evaluation of endotoxin levels included both bare MSP and MTX-loaded MSP after freeze-drying. For experimental purposes, the samples were reconstituted in endotoxin-free water at a concentration of 1.0 mg/mL. Concurrently, a standard curve was established using known endotoxin concentrations of 0, 0.005, 0.01, 0.025, 0.05, and 0.1 EU/mL. Absorbance readings were taken at 545 nm using the GloMax^®^ Discover Microplate reader (Promega, Sunnyvale, CA, USA). Utilizing the standard curve, the endotoxin levels of the samples were determined.

### 2.5. Particle Size, Surface Charge Analysis, and Morphology Studies

The particle size distribution of all samples was analyzed in MilliQ water using a Mastersizer^®^ 3000 Hydro MV (Malvern, United Kingdom), using refractive indexes of 1.33 and 1.52 for water and MSP, respectively. Specifically, the particles were dispersed in water, and each sample underwent 120 s of sonication before measurement to achieve a uniform resuspension without particle aggregation. The average particle size was expressed in terms of the volume median diameter d (0.9) [42].

The ζ-potential (PZ) of all samples was measured using dynamic light scattering (DLS) with a Zetasizer Nano 90ZS (Malvern Instruments Ltd., Worcestershire, UK) at 25 °C.

Size and charge measurements were conducted in triplicate, and the average value was reported. The morphology of all samples was characterized by optical, confocal fluorescence and scanning electron microscopy experiments.

A field-emission scanning electron microscopy (FEG-SEM, LEO 1530, Zeiss, Germany) was used to observe the MSP dispersed on an SEM sample stub covered with a silicon or graphite background.

Fluorescence microscopy experiments were carried out on an Axio-Zoom Scope microscope (Zeiss, Oberchoken, Germany), equipped with an HBO 50 Hg lamp and an AxioCam ICm1 CCD camera. Dry powdery samples were dispersed on glass slides.

Confocal laser scanning fluorescence microscopy (CLSFM) experiments were performed using an FV1000 (Olympus, Tokyo, Japan) confocal scanning device interfaced to an OlympusIX 81 inverted microscope. CLSFM measurements were carried out using an oil immersion 60× objective (numerical aperture: 1.35), and a 488 nm (Ar) and 543 nm (HeNe) laser for green (MTX) and red (MSP) channels, respectively. The 3D Volume rendering of the samples investigated were elaborated by Imaris 6.2.1 software (Bitplane, Switzerland). For this purpose, the HA polymer was labeled using the RBITC, as previously reported [43]. Briefly, the fluorescent dye RBITC was dissolved in MillliQ water at a concentration of 1 mg/mL [44]. After, 1 mL of the dye solution was added to the HA solution 1% (*w*/*v*) in a NaHCO_3_/Na_2_CO_3_ buffer (12 M, pH 9 [45]) and stirred for 12 h at room temperature in the dark. The resulted mix was then dialyzed against water (three times, 500 mL per time), using a dialysis membrane (MWCO 12–14 kDa Spectra/Por^®^), to allow the elimination of residual non-conjugated dyes [46].

### 2.6. In Vitro Release Profile of MTX from the Microsponge

MTX release from the MSP was performed using the dialysis method using a dialysis tube (MWCO 12–14 kDa SpectraPore, Spectrum Laboratories Inc., Rancho Dominguez, Canada) [47]. In particular, the lyophilized MTX-MSP was resuspended in 0.1 mL of PBS 1X (pH 7.4) and transferred into the dialysis membrane, which was previously soaked overnight in the dissolution medium (PBS, pH 7.4).

The membrane was tied from both ends and immersed in the PBS medium maintained at 37 ± 1 °C and stirred at 200 rpm. Aliquots were withdrawn periodically at a predetermined time interval (from 1 to 8 h each hour and after 24 h, up to 1 month), analyzed, and then replaced back into the release solution to maintain the volume constant [46]. To quantify the cumulative fraction of MTX released, withdrawn samples were assayed by measuring the absorbance at λ = 302 nm with a spectrophotometer, and using a standard curve of MTX concentration. Parallelly, the release profile of methotrexate was compared by placing the free drug inside the dialysis bag to evaluate its behavior under the same experimental conditions. Additionally, to demonstrate that the ability of the MSP to release MTX was not altered by the lyophilization step following loading, the release profile of the lyophilized sample was compared with that of the centrifuged sample, where the precipitate was resuspended in 0.1 mL PBS. All experiments were conducted independently in triplicate.

### 2.7. Ethical Approval

All experiments were carried out at the Interdepartmental Center for Comparative Medicine, Alternative Techniques and Aquaculture, University of Rome Tor Vergata. All the animal experiments were ethically approved by the Animal-Welfare body (OPBA) and authorized by the Ministry of Health, Legislative Decree no. 26/2014; European Directive 2010/63/UE (Authorization number 763/2021-PR—12 October 2021). This research was performed and reported according to the animal research: Reporting of In Vivo Experiments (ARRIVE) guidelines (https://arriveguidelines.org/, accessed on 1 October 2022).

### 2.8. Animal Husbandry

A total of 34 female and male Wistar rats aged 9 and 11 weeks (Envigo, Rome, Italy) were used in this study. The animals were acclimatized for 7 days before the experiment and housed under standard conditions: a 12/12-h light/dark cycle, room temperature of 20 ± 2 °C, and 55% relative humidity. The rats were provided with a standard laboratory diet and tap water ad libitum. A veterinary surgeon, responsible for the welfare of laboratory animals, was present during all experiments. Animal care was overseen by trained personnel. Rats were divided into groups, and the number of rats in each group was calculated using G Power Analysis.

### 2.9. Animal Treatment for Safety Study

In this study, both female and male rats were used. Rats were randomly divided into six groups (n = 3 per group), a control group and five treated groups of three animals each:

(i) Control group 0.8 mL of normal saline (NS) as vehicle;
(ii) MSP 1 mg/rat/0.8 mL in NS;
(iii) MSP 5 mg/rat/0.8 mL in NS;
(iv) Cross-linker 0.75 mg/rat/0.8 mL in NS;
(v) Cross-linker 3.75 mg/rat/0.8 mL in NS;
(vi) Hyaluronic acid 0.25 mg/rat/0.8 mL in NS.

To determine a safe concentration of the MSP, two doses were tested in this experiment: 1 mg and 5 mg. Additionally, the individual components of the MSP, CL, and HA were also evaluated. The quantities of these individual components were calculated based on the ratio of CL to HA in the MSP, which was approximately 1:4. For hyaluronic acid, only one dose, equivalent to 1 mg of MSP, was selected, because according to the literature data, the widely used hyaluronic acid generally does not exhibit significant toxicological effects, and its safety profile is well established and recognized [48]. By excluding this group, we ensured compliance with ethical standards while maintaining the integrity and relevance of the study’s findings. Instead, regarding the cross-linker, both doses, corresponding to the amounts present in 1 mg and 5 mg of MSP, were evaluated.

**Figure 1:**
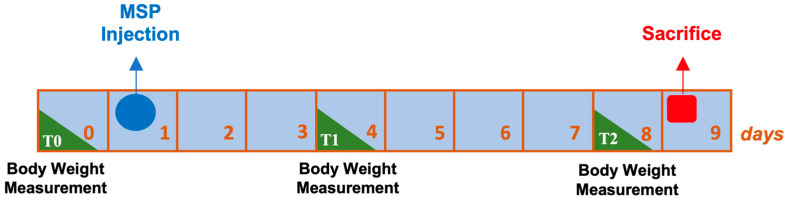
Experimental design of safety study.

All animals received a single subcutaneous injection on day one of the experimental plan (Figure 1). The body weight measurement took place at T0, which corresponds to the day before the administration of the MSP, at T1, which is the fourth experimental day, and at T2, the eighth day of the experimental plan that preceded the sacrifice. On the ninth day post-injection, the rats were sacrificed, and histopathological studies were conducted.

### 2.10. Animal Treatment for Efficacy Study

**Figure 2:**
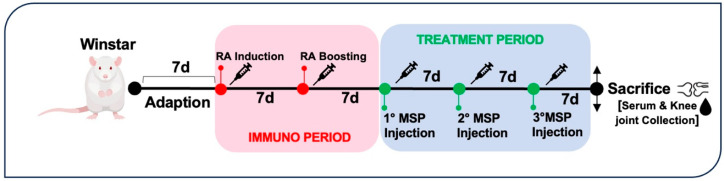
Experimental design of efficacy study.

Seventeen rats were randomly divided into five groups. Four groups were immunized intradermally with 200 μg Type II collagen from bovine tracheal cartilage (Sigma C1188) in 0.05 M acetic acid/rat/100 μL emulsified with an equal volume of incomplete Freund’s adjuvant (IFA) (Chondrex, Redmond, WA, USA) on day 7 to induce RA. A boost injection of 100 μg collagen-IFA suspension was given in the same manner on day 15 (Figure 2).

In the first experiment conducted, the animals were randomized into the following experimental groups:

(i) Healthy non-arthritic as negative control (3 rats);
(ii) Arthritic untreated as positive control (4 rats).

From day 22 to 42, immunized groups were subcutaneously administered with a single injection every week of the following:

(iii) MTX 0.125 mg/rat in 0.1 mL NS for the MTX group (3 rats);
(iv) MSP 1 mg/rat in 0.1 mL NS; for the MSP group (4 rats);
(v) 1 mg/rat in 0.1 mL NS; for the MTX-loaded MSP lyophilized group (3 rats).

Meanwhile, in the second study, the experimental design included the following:

(i) Positive control group: untreated arthritic rats;
(ii) MSP group: arthritic rats treated with 1.5 mg MSP/rat in 0.1 mL NS;
(iii) MTX group: arthritic rats treated with 0.125 mg MTX/rat in 0.1 mL NS;
(iv) MTX-MSP centrifuged group: arthritic rats treated with 1.5 mg MTX-MSP centrifuged/rat in 0.1 mL NS;
(v) MTX-MPS lyophilized group: arthritic rats treated with 1.5 mg MTX-MSP lyophilized/rat in 0.1 mL NS.

In the design of the second experiment, variations were made to the groups; specifically, a higher amount of MSP (1.5 mg) was used while keeping the loaded methotrexate concentration constant, and both the centrifuged and lyophilized samples were evaluated.

All animals were anesthetized with Ketamine 70 mg/kg and Medetomidine Chlorhydrate 0.1 mg/kg and sacrificed by intracardiac blood sampling with subsequent aortic arch recession on day 43. In the first experiment, both posterior knees and serum were collected for analysis while in the second experiment, only the right knees were collected. For each rat, an aliquot of about 1 mL of blood was used for obtaining the serum, and an aliquot of about 0.5 mL of blood was used to make the blood cell count.

### 2.11. Measurement of Hematological Parameters

For determination of hematological parameters, 20 µL of whole blood, collected in K2EDTA microtainers (Becton, Dickinson and Company, Franklin Lakes, NJ, USA), was analyzed using the commercially available automated cell counter “Drew3” (BPC BioSed s.r.l., Castelnuovo di Porto RM, Italy). For cytomorphological examination, each sample from peripheral blood smears was prepared using the differential staining Diff-Quick (Dade SpA, Milan, Italy) and analyzed under optical microscopy. The complete blood count (CBC) includes the following: haemoglobin (HGB), haematocrit (HCT), red blood cell (RBC) count, white blood cell (WBC) count, platelet count (PLT), red cell distribution width (RDW), mean corpuscular volume (MCV), mean platelet volume (MPV), mean corpuscular volume (MCV), mean corpuscular haemoglobin (MCH), mean corpuscular haemoglobin concentration (MCHC), red cell distribution width (RDW), lymphocytes (LYMF), mid-range cells (MID), granulocytes (GRAN), platelet distribution width (PDW), platelet large cell ratio (LPCR), and plateletcrit (PCT).

### 2.12. Detection of Spleen Index and Thymus Index

After the measurement of rat weight, rats were sacrificed. The spleen and thymus were isolated from rats and weighed immediately. Thymus index and spleen index were analyzed based on the following equation: thymus index or spleen index = (weight of thymus or spleen)/body weight.

### 2.13. Histological Analysis

For the toxicological study, the lung, liver, kidney, and spleen were formalin fixed and paraffin embedded. Architectural modification, as well as the presence of inflammatory infiltrate, were evaluated on a 4-µm serial section stained with haematoxylin and eosin (H&E) (see Table 1). Right hind limbs were formalin fixed, then decalcified in 0.5 M EDTA and 0.5% paraformaldehyde for 24 h. Decalcified samples were dehydrated on an alcohol series, cleared with xylene, and paraffin embedded. Five-micrometer thick sections of talocrural joint sections were stained with H&E and mounted on glass slides for histological analysis by light microscopy. Histopathological changes of the joint were evaluated based on morphological parameters (structure, cells, matrix, and tidemark) that were previously reported [49]. Specifically, we used a cumulative score ranging from 0 to 11 points. Higher scores indicate greater tissue damage. The criteria used to evaluate the histopathological score are shown in Table 2.

### 2.14. Cytokine and Antibody α-Collagen ELISA Assays

IL1-β quantification in rat serum was performed using an ELISA kit (BMS630, Invitrogen, Thermo Fisher Scientific, Inc., Waltham, MA, USA) according to the manufacturer’s instructions.

Antibody levels to type II collagen were measured using an enzyme-linked immunosorbent assay (ELISA) technique. Next, 96-well plates were coated with type II collagen (C1188; Sigma-Aldrich, St. Louis, MO, USA) diluted (final concentration 2 µg/mL) in 100 µL of phosphate buffer (pH 9.3), then incubated overnight, washed three times and blocked using PBS containing 1% bovine serum albumin (PBS-BSA) for 1 h. The sera were incubated (37 °C, 1 h) with 100 µL of serum (1:100). After the incubation with sera, all plates were incubated (37 °C, 1 h) with anti-rat IgG secondary antibody, and were HRP-conjugated (Invitrogen, Milan, Italy). The absorbance (OD) at 495 nm of antigen- and buffer-coated wells was measured, and the difference in mean OD values was calculated. All samples were assayed in duplicate to increase precision.

### 2.15. Statistical Analyses

Experiments were repeated three times. Data were reported as the mean values ± standard error of the mean (SEM) or standard deviation (SD).

To calculate the size of each in vivo treatment group for preclinical safety and efficacy studies, G*Power 3.1 power analysis software was used, considering an 80% probability of detecting the difference and α error of 0.05. This analysis ensured both the reliability of findings and ethical minimization of animal use.

For the analysis of data derived from in vitro experiments, differences in means were assessed for statistical significance using ANOVA and Tukey tests, with *p* values < 0.05 considered significant.

Statistical comparisons between animal groups were performed using a 2-Way ANOVA, with *p* values < 0.05 considered to be statistically significant.

GraphPad Prism version 7.0 statistical software (GraphPad Software Inc., San Diego, CA, USA) was used for all statistical analyses and graphs.

## 3. Results and Discussion

This study was designed as a proof-of-concept preclinical trial aimed at evaluating the safety and efficacy of the MSP. In particular, the focus of this investigation was the use of MSP technology to deliver methotrexate, a drug currently used in the management of RA, via subcutaneous administration.

### 3.1. Physicochemical Characterization of Unloaded and Loaded Microsponge

Hyaluronic acid-based MPS were synthesized using an established one-pot self-precipitation/cross-linking method [32,37]. In contrast to previous studies, the hyaluronic acid utilized in this work had a higher molecular weight (1590 kDa) compared to the reported ranges (20–70 kDa [43] and 1200 kDa [50]). However, the synthesis steps remained unchanged to maintain the patented platform. This choice was based on the literature, suggesting that high molecular weight HA exhibits anti-angiogenic and anti-inflammatory properties [51].

**Figure 3:**
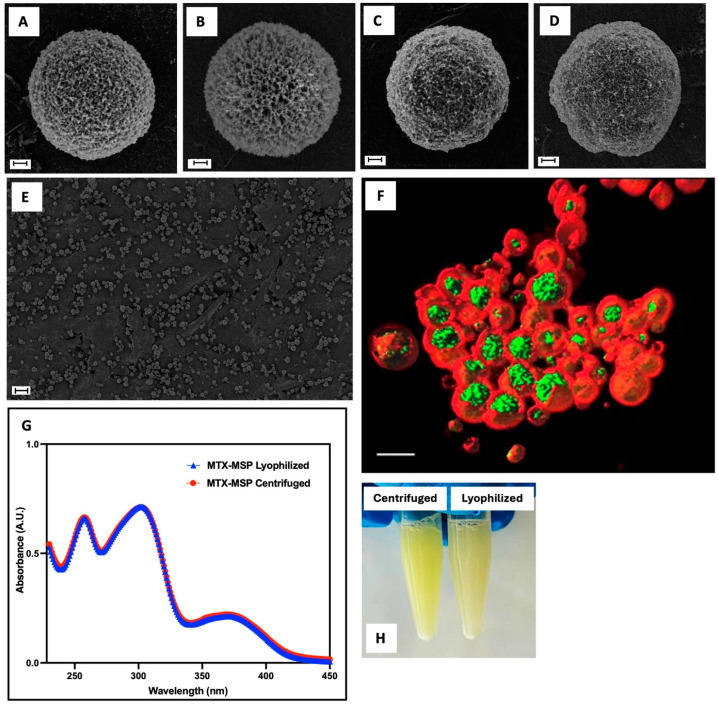
SEM images of the MSP before (Panel (**A**)) and after (Panel (**B**)) the freeze-dry step; MSP loaded with MTX after centrifugation (Panel (**C**)) and after freeze-drying (Panel (**D**)); scale bar: 1 μm; morphology of the MTX-MSP population by SEM (Panel (**E**); scale bar: 20 μm) and by confocal analysis (Panel (**F**), 3D rendering with isosurfaces where green color indicates MTX, and red color indicates MSP; scale bar: 5 μm)). MTX spectra of MTX-MSP by UV-Vis after centrifugation and lyophilization processes (Panel (**G**)). Picture of the MSP loaded with MTX, centrifuged and lyophilized, after reconstitution with a PBS buffer (Panel (**H**)).

After synthesis, all formulations were freeze-dried, converting the suspension into a powder. A well-designed freeze-drying cycle ensures both the physical and chemical stability of the final formulation, thereby prolonging its long-term conservation. It also allows for adjusting concentration and the dispersant type during reconstitution, facilitating easy redispersion for parental administration. Generally, the freeze-drying process involves (i) initial freezing of the suspension, (ii) primary drying, during which water is removed from the formulation by sublimation without passing through a liquid phase, and (iii) secondary drying, which removes any adsorbed surface-bound water and reduces residual moisture content, resulting in a free-flowing dry powder. However, a drug delivery platform may be damaged during lyophilization and/or subsequent rehydration if appropriate stabilizers are not used. Such instability can lead to aggregation and coalescence of the MSP into significantly larger constructs [47]. Cryoprotectants were not used in the MSP formulation because the SEM analysis (Figure 3) revealed no evidence of morphological instability. This is evident by comparing panels A and B, for the empty MSP and panels C and D, for the loaded MSP. The result was confirmed by particle size analysis using the same technique (Table 3), showing no significant changes between filled and empty samples, before and after lyophilization. Moreover, all synthetized MSP displayed a spherical shape of a nano-sized porous surface with a homogenous population (Figure 3, Panel E).

To achieve effective loading of MTX on the porous surface of the MSP through physical absorption, lyophilized MSP were exposed to a MTX solution. For drug loading studies, the lyophilized formulation was used because according to Crotts and Park [52], the lyophilization process leaves pores open and available for drug loading by removing solvent (water).

Absorbance measurements were taken on centrifuged samples at different time intervals to determine the time required to achieve maximum drug absorption.

**Figure 4:**
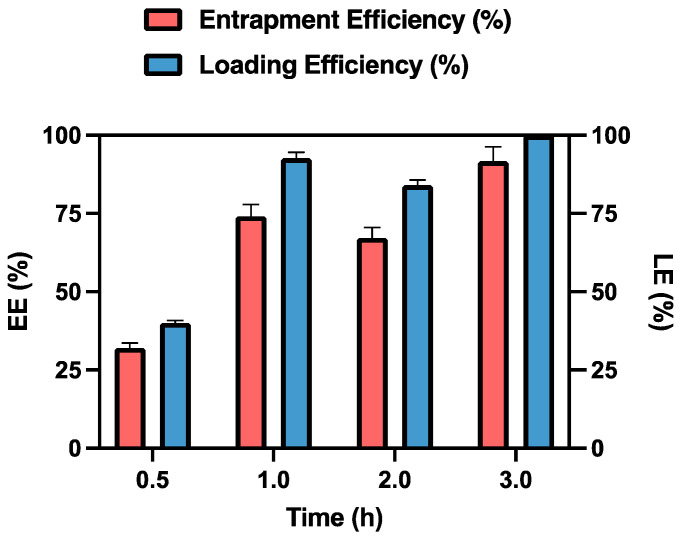
Entrapment Efficiency (EE%) and Loading Efficiency (LE%) of MTX-MSP over time. Results are presented as the average ± standard deviation (SD) (n = 3).

As shown in Figure 4, after 3 h of contact, the MSP retained 91.76% of the initially loaded dose, expressed as entrapment efficiency, which corresponds to 0.125 mg of MTX. This result is promising for the in vivo studies, as the recommended weekly dose of MTX for humans is 7.5–15 mg [53]. When adjusted for rats, the equivalent acceptable range for this study is 0.11–0.23 mg/week, based on dosage conversion criteria between animals and humans [54].

After 24 h, the data remained unchanged, which is why 3 h was established as the optimal time for drug loading.

Analysis of the loading capacity of the MSP for MTX at the used concentration reveals that it is approximately 110% at the 3-h mark. This result indicates that 1 mg of MSP can effectively entrap the loaded MTX. The observed excess in the maximum amount of loaded capacity can be explained by the fact that the amount of MSP used in this experiment is fully capable of accommodating the MTX within the MSP pores [40]. In fact, when using 10 mg of MSP while maintaining a constant MTX concentration, the loading efficiency percentage decreases.

This finding is also confirmed by the data obtained by fluorescence confocal microscopy (Figure 3, Panel F). The image is acquired in xyz mode and 4× optical zoom and is shown in volume rendering with isosurfaces for the green and red channels. It shows that MTX (in green) is primarily localized within the pores of the MSP (in red). Additionally, MTX absorption appears homogeneous across the entire population of the MSP, as shown in Figure S1 (Supplementary Materials). The confocal images reveal that MTX (in green, Panel B) is uniformly distributed within the MSP (in red, Panel A) across all particles. The merged image (Panel C) clearly demonstrates the thorough and consistent localization of the drug within the MSP pores. Since drug loading is achieved through physical adsorption, the interaction between MTX and the MSP is crucial to ensure strong drug–support interactions.

According to a recent study based on computational methods [55], the MTX absorption on the HA surface within the MSP pores is driven primarily by a combination of polar covalent bonding and electrostatic interactions. These forces are most effective in aqueous environments, where the negatively charged carboxylate groups of HA play a critical role in binding MTX, which contains both charged and polar regions. Specifically, the electrostatic interactions arise from ionic bonds formed between the negatively charged carboxyl groups of HA and the positively charged amine groups of MTX. Moreover, hydrogen bonding and hydrophobic interactions may also contribute to the stabilization of the MTX-HA complex within the MSP pores, enhancing drug retention [56]. These interactions become particularly significant in the physiological context of RA, where factors such as pH and ionic strength could influence the binding and retention of MTX within the matrix.

In a prior study by our group [43], we found that pH significantly influences the adsorption and release behavior of biomolecules and therapeutic agents within MSP. Proteins like lysozyme and BSA showed pH-dependent retention due to their isoelectric points (pI ≈ 11 and 4.7, respectively). At acidic pH, stronger electrostatic interactions slowed their release, while at higher pH, the reduced interactions facilitated faster release.

MTX, however, behaves differently due to its specific functional groups. MTX contains carboxylic acids (pKa ≈ 3.8, 4.8) and an amine group (pKa ≈ 5.6). At pH 5, it adopts a zwitterionic form, where partially deprotonated carboxylic groups (negative) and protonated amine groups (positive) promote electrostatic and hydrogen bonding with the matrix. This slows its release, making mildly acidic environments like inflamed tissues in RA ideal for sustained drug delivery [56,57]. At physiological pH (≈7.4), the carboxylic acids are fully deprotonated, and MTX becomes predominantly negative, weakening interactions with the matrix. This leads to faster release kinetics, aligning with reduced retention in neutral environments [58]. These findings highlight that while pH-sensitive systems benefit both protein-based and small-molecule therapeutics, the underlying mechanisms differ. Moreover, for MTX, the pKa-driven behavior suggests the opportunity to optimize functional groups to enhance controlled delivery and therapeutic targeting in specific physiological conditions.

The sample was subsequently freeze-dried, a common pharmaceutical technique for stabilizing drugs, vaccines, antibodies, and biological materials [59]. According to the literature, this process preserves the active pharmaceutical ingredient (API) and its activity. However, it does not guarantee that the physical state of the final formulation remains unchanged [31]. To ensure that not just the morphology and size were unaltered by this technique but that also the drug itself was stable, the UV-Vis MTX spectrum was analyzed. As shown in Figure 3, Panel G, no significant changes were observed in MTX spectra, confirming the stability of the MTX-MSP formulation after freeze-drying. Additionally, Panel H (Figure 3) shows that the formulation can be easily reconstituted within seconds, indicating no particle aggregation [39].

Table 4 shows the average particle size, expressed as 90th percentile, and ζ-potential values of empty MPS and MTX-MPS reconstituted after lyophilization.

Photon correlation spectroscopy revealed a particle size d (0.9) of 34.5 µm for the empty MSP resuspended after freeze-drying. After the addition of MTX, the particle size significantly increased to 60.8 µm, indicating successful drug loading into the MSP [60].

The ζ-potential values were measured as −36 mV for empty MSP and −43 mV for MTX-MSP. According with the literature [61], particles with a ζ-potential higher than +30 mV or less than −30 mV typically exhibit steric stabilization due to electrostatic repulsion forces, which prevent agglomeration. This suggests that the achieved ζ-potential values ensure steric stabilization and maintain the physical stability of the formulation. These findings indicate that both the composition and synthesis processes used in this work make the samples electrically stable. Additionally, the negatively charged particles demonstrate enhanced lymphatic uptake and prolonged retention time [61].

Manufactured samples must be free of microbial agents to protect tissues and reduce patient inflammatory and immune responses. Microbial agents can be introduced through raw materials or the manufacturing process. To ensure the absence of such agents, all samples were tested for endotoxin contamination. Endotoxins, which are lipopolysaccharides (LPS) from Gram-negative bacteria, are a common cause of toxic reactions due to pyrogens. Endotoxin assays test for viable Gram-negative bacteria and also detect LPS from dead bacteria. Therefore, the absence of LPS indicates an absence of pyrogens. In this study, samples were tested for endotoxins using a Limulus Amebocyte Lysate (LAL) assay. The results, shown in Table 4, indicated that each sample contained very low levels of endotoxin in each sample (below 0.01 EU/mL) [62].

This is significantly lower than the threshold for being considered endotoxin-free. According to regulatory standards, the threshold for endotoxins in SC drug formulations is typically set at 0.5 EU/mL, which warrants the minimization of pyrogenic reactions, such as fever or shock, in patients receiving the treatment [63,64]. These results confirm the suitability of our formulations for injection, ensuring their safety and efficacy for this route of delivery.

### 3.2. MTX Release Studies from the Microsponge

The in vitro release of MTX from the loaded MSP was evaluated using a dialysis membrane and compared to the release profile of the free drug. Additionally, to assess whether the lyophilization technique altered the release capacity of the platform, release profiles of MTX from both lyophilized and centrifuged formulations were evaluated and compared.

**Figure 5:**
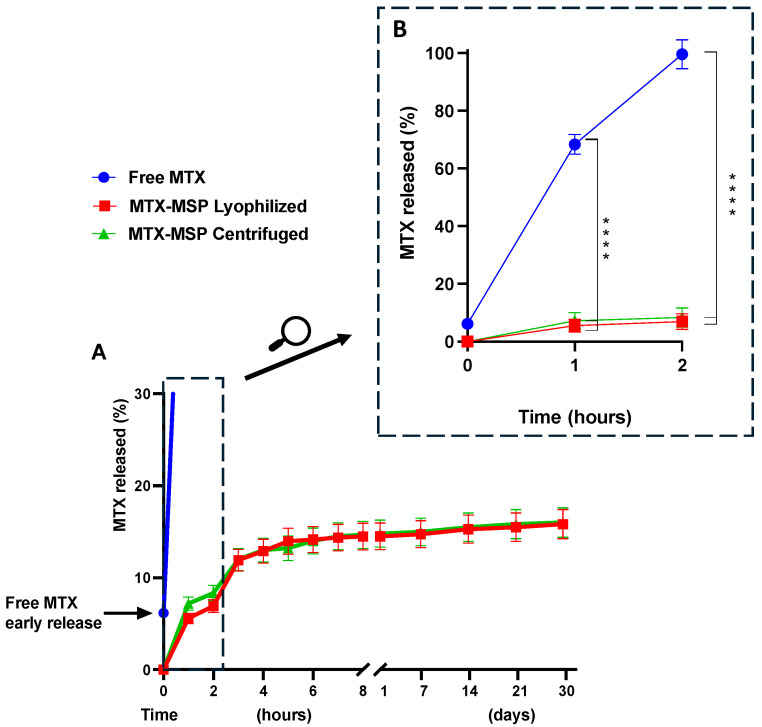
Methotrexate release profile. Panel (**A**): comparison of the MTX release percentage among free MTX, lyophilized MTX-MPS, and centrifuged MTX-MPS over a period of one month. Panel (**B**): enlargement of the section depicting the release profile within the first 2 h for the three formulations. The results are expressed as mean ± standard deviation and were performed in triplicate. **** *p* < 0.0001 compared with Free MTX.

As shown in Figure 5, Panel B, the conventional formulation (free MTX) was immediately released into the external medium upon immersion of the dialysis bag (time 0), followed by the release of 50% of the drug within 30 min and complete exhaustion within 2 h.

In contrast, both MSP formulations exhibited a very slow initial burst release (Figure 5, Panel B), with a significant decrease in release compared to free MTX, as indicated by the 95% confidence interval and a *p*-value of <0.0001 (see Supplementary Materials, Figure S2). This was followed by a sustained release phase lasting over 30 days (Figure 5, Panel A). Moreover, the freeze-drying step did not significantly modify the release profile of MTX.

The absence of an initial burst release at the time zero from the formulation confirms the absence of non-encapsulated MTX on the exterior of the MSP. The MTX release reached about 14.51% during the first few hours of the experiment, followed by a drastic drop in release-rate after 3 h, indicating the onset of prolonged drug release from the MSP, thereafter, lasting up and beyond 1 month. Initially, the rapid release of the drug from the MSP could be attributed to the superficial MTX adsorbed on the porous surface, while the sustained release suggests that most of the drug is retained within the nano-sized pores [65]. Furthermore, the drug release from the MSP is also controlled by interactions between MTX and hyaluronic acid. In fact, the slow release of the drug from the MSPs can be explained by the results obtained by Faizul Azam et al. [55]. By all-atom molecular dynamics simulations, they showed that the HA-MTX complex is extremely stable in an aqueous solution, preventing the premature release of MTX immediately after resuspension at the time of administration.

These findings support that the developed hyaluronic acid-based MSP are a promising drug platform for MTX in RA applications and possibly beyond.

### 3.3. In Vivo Safety Study

As an initial step toward a preclinical evaluation, an in vivo safety experiment was conducted to evaluate the potential effects on target organs following a single injection of empty MSP and its individual components in a healthy animal model using both male and female Wistar rats. The study assessed the effect of a single subcutaneous dose of MSP on the structure and function of the liver, lungs, kidneys, and spleen of the rats. At sacrifice, the blood, liver, kidneys, spleen, and lungs samples were collected for histological examination.

As shown in Figure 1, animals were weighed three times, (i) before the administration of the MSP—T0, (ii) on day 4 after MSP administration—T1, and (iii) the day before sacrifice—T2.

**Figure 6:**
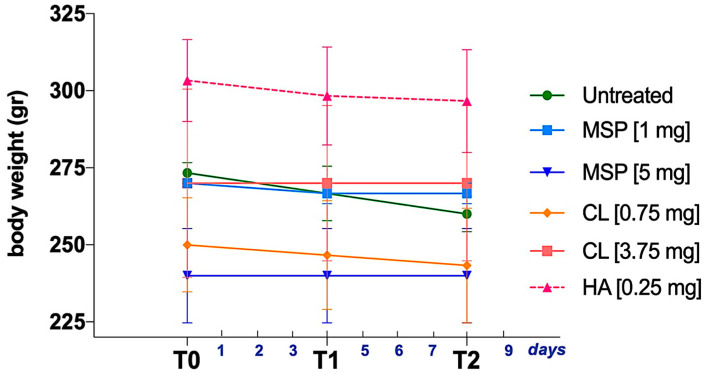
Body weight measurement at three key experimental time points: T0 (the day before MSP administration), T1 (the fourth experimental day), and T2 (the eighth experimental day, preceding the sacrifice). Data are presented as mean ± SD.

No significant body weight differences were observed between experimental and control groups (Figure 6). No adverse effects on body weights were noted in either sex throughout the study. No clinical signs of morbidity or mortality were observed during the study.

**Figure 7:**
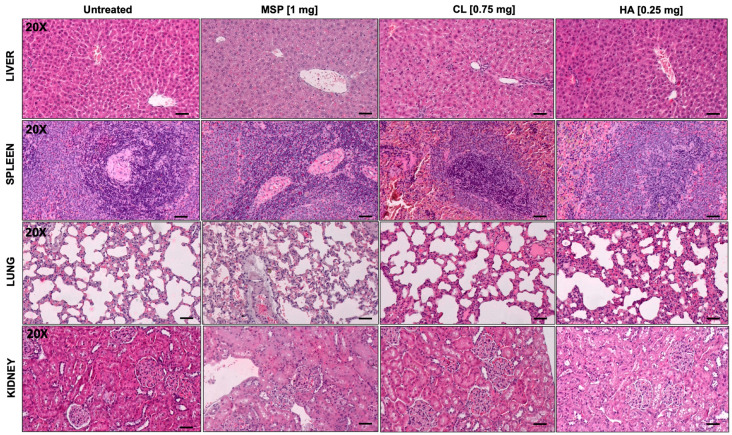
Histopathological analysis for safety study. Histological analysis of knee joint sections by H&E staining: liver; spleen; lung; kidney. 20× magnification: Scale bar = 50 μm.

**Figure 8:**
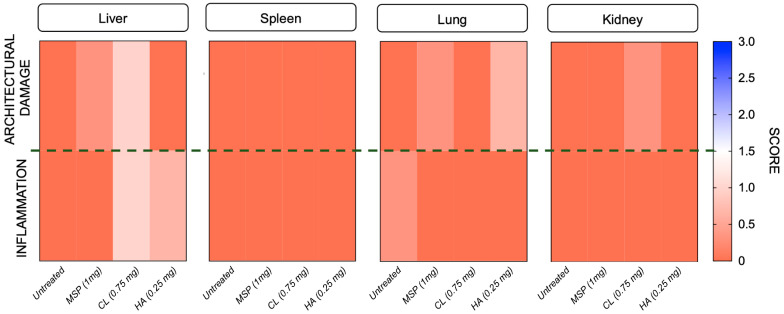
Heat map illustrating Architectural Damage and Inflammation Scores for each tested organ. In the heat map, orange indicates lower scores, while blue represents higher scores.

Blood count parameters (Table S1, in Supplementary Materials) and histopathological evaluations (Figure 7 and Figure 8, Figures S3 and S4) displayed no anomalies. In particular, no significant macroscopic differences were observed at necropsy, and no macroscopic or microscopic changes were reported.

Any histopathological change in examined organs did not reach statistical significance. Based on a combined score of architectural damage and inflammation (Figure 8 and Figure S4), histopathological evaluation revealed in fact no abnormalities related to the subcutaneous injection of the MSP or its components.

In conclusion, the single subcutaneous administration of two different doses of MSP (1 mg and 5 mg), and their respective components (CL and HA), was well tolerated in rats and caused no adverse clinical or anatomical pathology findings, proving the safety of the DDP.

### 3.4. In Vivo Efficacy Study

**Figure 9:**
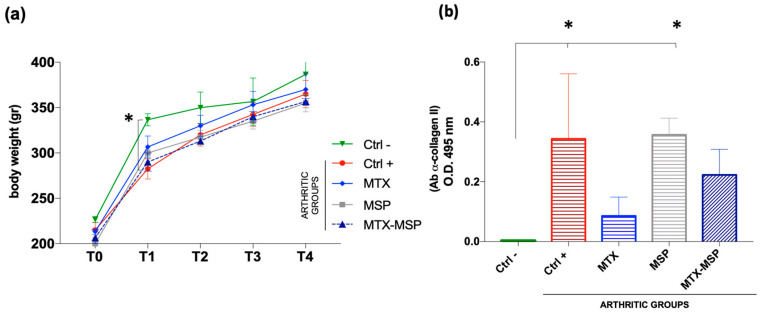
(**a**) Body weight measurement; (**b**) ELISA assay against Ab α-Collagen II. Data are presented as mean ± SD. * *p* < 0.05 compared with negative control.

Following the safety study, encompassing doses from 1 mg to 5 mg of MSP, we proceeded to test for efficacy by conducting a first experiment with about 1 mg of MSP loaded with 0.125 mg of MTX. The study investigated the efficacy of weekly subcutaneous treatment with the MTX-loaded MSP compared to MTX alone, in the CIA rat model. At sacrifice, blood samples and knee joints were collected (Figure 2). The therapeutic efficacy of the MTX-loaded delivery platform was evaluated in terms of the histopathological RA score, a key parameter for assessing the anti-inflammatory effect. This specific histological RA score was evaluated for both posterior knees of the CIA animals. Similar body weight trends were observed in rats from several treated groups compared to the positive and negative control groups, without significant variations (Figure 9, Panel a).

To confirm RA induction in the positive control group and study post-RA induction changes in rats, the expression levels of autoreactive antibodies against CII (CII-specific IgG) were evaluated in the serum samples collected at the end of the experiment. The Anti-CII antibody levels in the positive control group and the empty MSP group were significantly higher than those in the negative control group (Figure 9, Panel b).

The results suggest that treatments with free MTX and the MTX-loaded MSP positively affected the pathology, influencing the levels of expression of antibodies against CII. Though not statistically significant compared to the positive control, a clear downward trend in antibody expression levels was observed in the treated groups, hinting to an improvement in therapeutic efficacy of MTX-MSP.

**Figure 10:**
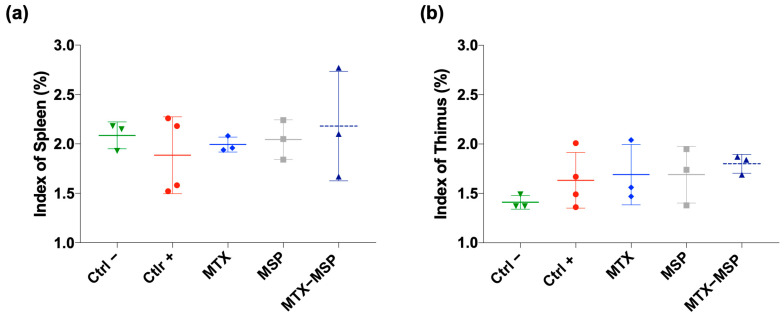
Index of spleen and thymus at sacrifice for all groups. Data presented as mean ± SEM.

To assess the impact of treatments on immune organs, the weights of spleen and thymus were measured at the end of the experiment to calculate thymus and spleen indices, respectively. The results indicate that all treatments had non-significant effects on both indices in comparison to both negative and positive control rats. Differences between groups were not statistically significant (Figure 10, Panel a and b).

**Figure 11:**
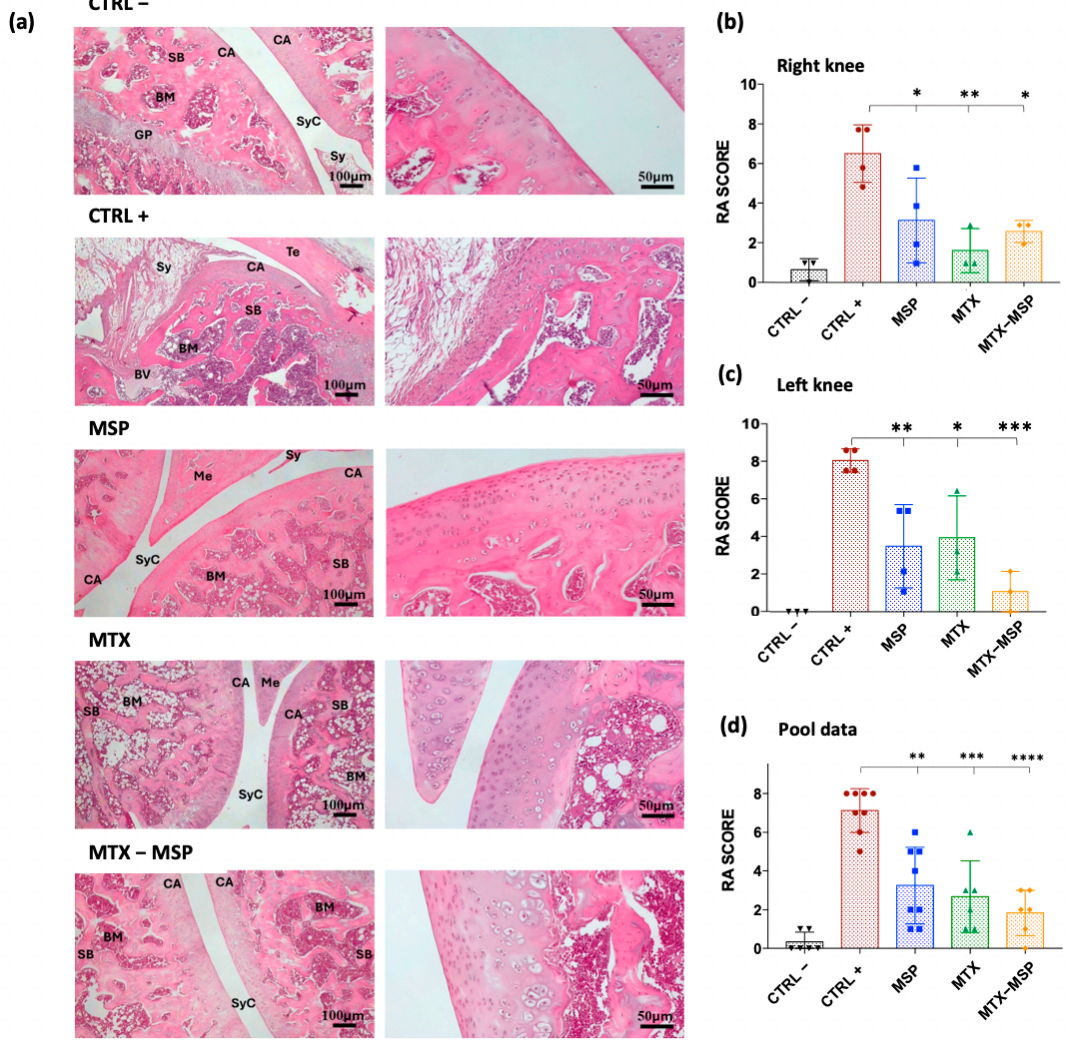
(**a**) Histological results of the knee joints stained with hematoxylin and eosin. BM: bone marrow; BV: blood vessel; CA: cartridge; GP: growth plate; Me: meniscus; SB: subchondral bone; Sy: synovia; SyC: synovial cavity; Te: tendon. (**b**) Rheumatoid Arthritis scores of rats in different groups for both analyzed knees; (**c**) Rheumatoid Arthritis scores of rats in different groups for right analyzed knees; (**d**) Rheumatoid Arthritis scores of rats in different groups for left analyzed knees. Data are presented as mean ± SD. * *p* < 0.05; ** *p* < 0.01; *** *p* < 0.001; **** *p* < 0.0001 compared with positive control.

To evaluate the impact of weekly MTX-loaded MSP subcutaneous treatment on joint destruction, both posterior joints were analyzed histologically (Figure 11, Panel a).

**Figure 12:**
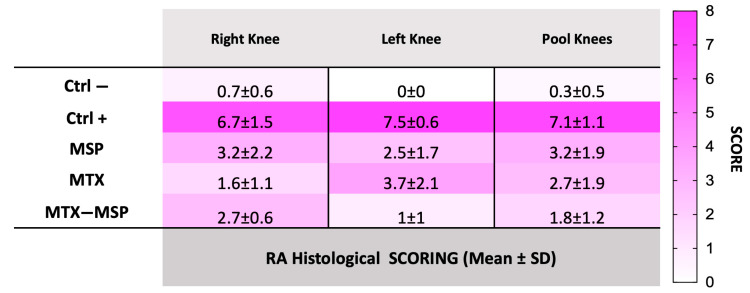
RA histological scores for the right knee, left knee, and pooled knees under various conditions. Scores range from 0 to 8, with darker shades indicating higher scores. Data are presented as mean ± SD.

H&E staining of both hind limbs in the negative control group revealed normal structure, normal cellular organization, matrix staining, and tidemark integrity. Conversely, as expected, the analysis of RA rats in the positive control group showed structural irregularities, cellular clusters, reduced matrix staining, and altered tidemark integrity. These modifications were reflected in increased RA score in both limbs (Figure 11, Panel b). The comprehensive tables detailing the data for all categories analyzed in this study, including cells, tidemark, structure, and matrix, are available in the Supplementary Materials. The scores from these individual categories were aggregated to derive the final RA histological score. Specifically, the mean values used for calculating the RA score are presented in Tables S2–S4 (in the Supplementary Materials). The histological RA scores for each treatment group are presented in Figure 11, which includes pooled data from both knees (Panel b), as well as separate scores for the right (Panel c) and left limb (Panel d). Moreover, the data illustrated in the histograms of Figure 11 are also represented in the Figure 12, which includes a colorimetric grading scale. This scale visually demonstrates the reduction in the severity of the pathology at the histological level, indicated by a lighter pink coloration. This visual representation facilitates an intuitive understanding of the decrease in disease severity.

As shown in Figure 11, Panel b, the treatment with the MSP alone significantly reduces the RA score, and an even more pronounced reduction was observed in the group treated with MTX alone. The MTX-loaded MSP group, however, exhibits the most significant reduction in this histological scoring.

Figure 11, Panel c shows that all treatments significantly impact the reduction of the RA score in the right knees, with the MTX alone group showing the greatest reduction. Figure 11, Panel d presents the results of the histopathological analysis of the left knees. The MTX-MSP lyophilized group achieves a significant reduction compared to the control group, although significant reductions are also evident in the other two groups.

The beneficial histological effect observed with the MSP alone may be attributed to the high molecular weight of the hyaluronic acid used in the platform synthesis, which is known to significantly affect inflammation associated with RA [51,66]. This result confirms that selecting 1 mg of MSP is sufficient not only for effectively trapping the loaded MTX but also for having a significant biological effect associated with the presence of hyaluronic acid.

During standard subcutaneous (SC) administration, the MTX formulation is injected into the epidermal and dermal layers, reaching the subcutaneous fatty tissue [67]. From there, it enters the bloodstream via capillaries or the lymphatic system, depending on the molecular size of the therapeutic agent. Unlike intravenous injection, the SC route requires absorption, resulting in a slower systemic uptake. The MSP formulation, once injected, forms localized depots in the fatty tissue due to their micrometric size [68,69,70], which contributes to extend the timescale of MTX absorption, providing prolonged exposure while reducing peak plasma concentrations and minimizing the fluctuations seen with conventional MTX formulations. However, the precise mechanisms underlying MSP behavior and degradation post-injection require further investigation.

In line with the enhanced efficacy observed for the MTX-MSP combination, the degradation profile of the MSP in vivo likely contributes to the sustained therapeutic effects. As shown in our previous study [50], MSP degrade, in fact, via both redox and enzymatic pathways. Redox disassembly occurs efficiently within hours, while enzymatic degradation is slower due to the cross-linked structure of the MSP, which limits enzyme access. This slow degradation mechanism may contribute to the extended presence of MSP in the site of administration, resulting in prolonged drug release and sustained therapeutic effects, thus enhancing the efficacy of the MTX-MSP treatment over MTX alone.

**Figure 13:**
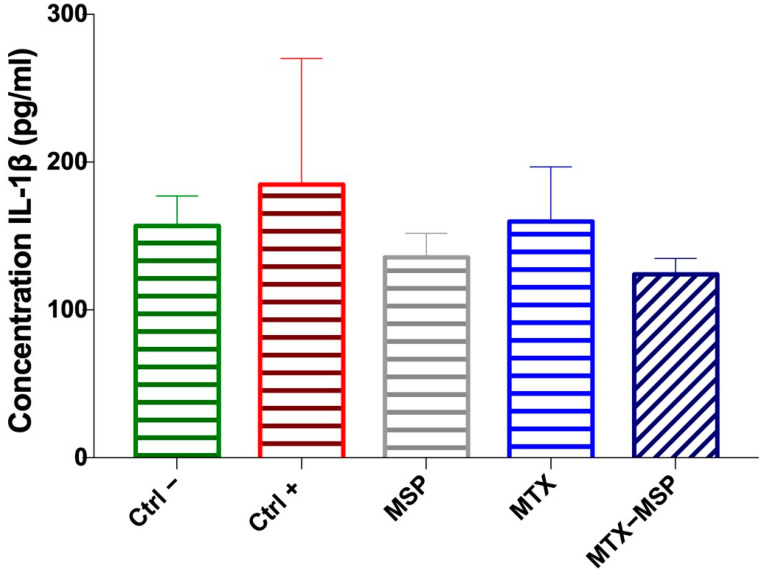
The levels of IL-1β cytokine from serum determined by ELISA assay at the end of the experiment. Data are presented as mean ± SD.

Additionally, the expression levels of IL-1β were measured in the serum collected at the time of sacrifice. Although no significant decreases were observed, a clear downward trend in IL-1β expression levels compared to the positive control group was evident. In fact, the expression levels of this cytokine in the groups treated with the empty MSP and the MTX-loaded MSP returned to similar values as those in the negative control group, albeit with a slight but notable reduction, especially in the MTX-loaded MSP group (Figure 13).

The CBC results (detailed in Table S5—Supplementary Materials) show that all treatments had a similar impact on blood parameters, with no abnormalities or significant deviations observed. Key hematological parameters, including WBC, RBC, hemoglobin, hematocrit, MCV, MCH, MCHC, platelet count, and differential leukocyte counts, remained stable across treatment groups. These findings indicate that MTX alone, the MSP alone, and their combination did not negatively affect hematopoiesis or blood health. The results align with histopathological data (from Tables S2 to S4—Supplementary Materials), supporting the hematological safety of the treatments. The absence of significant changes in blood parameters suggests that the treatments were well tolerated and did not exacerbate RA, nor cause the adverse hematological effects commonly linked to inflammatory conditions.

**Figure 14:**
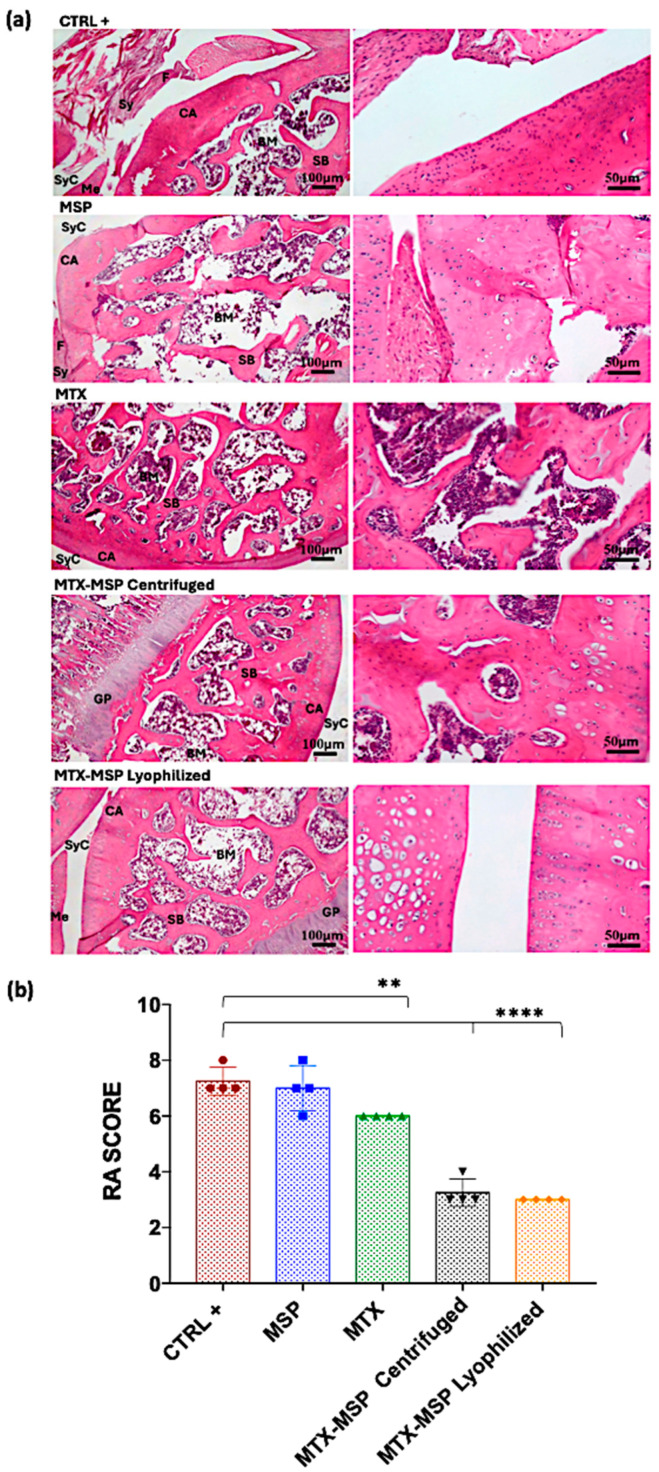
(**a**) Histological results of the knee joints stained with H&E; BM: bone marrox; CA: cartridge; F: fibrin; GP: growth plate; Me: meniscus; SB: subchondral bone; Sy: synovia; SyC: synovial cavity; (**b**) Rheumatoid Arthritis scoring of rats in different groups for analyzed right knees. Data are presented as mean ± SD. ** *p* < 0.01; **** *p* < 0.0001 compared with positive control.

The results from the initial preclinical study prompted a second confirmatory experiment that was slightly redesigned by increasing the MSP dose to 1.5 mg while maintaining the MTX concentration. The purpose of this change was to determine whether a higher MSP dose would enhance the biological effect in the CIA rat model and to probe the robustness of the proposed approach. Additionally, to ensure that the biological outcomes were not influenced by the DDP preparation technique, we also evaluated efficacy using centrifuged MTX-MSP, i.e., where an empty MSP platform was provided as dry powder, and the MTX liquid drug was loaded in it at the time of injection by simple mixing. Conversely, in the lyophilized MTX-MSP, both the drug and the platform were pre-lyophilized and provided as a preloaded powder ready for use off-the-shelf at the time of injection. H&E staining analysis (Figure 14, Panel a) revealed that both treatment groups exhibited significantly lower histological RA scores compared to the positive control group (Figure 14, Panel b).

The data from this second animal set confirmed the results from prior experiment, verifying that, while standard MTX treatment is effective (i.e., a significant reduction in the RA score exists, Figure 14, Panel b), combining MTX with our MSP drug delivery platform substantially enhances the therapeutic effect. Contrary to the results obtained in the first experiment, though, increasing the MSP dose alone did not lead to a significant enhancement of the biological effect, indicating that the therapeutic benefits are due to the combination of the platform with MTX.

### 3.5. A Clinical Perspective

The MSP system bears the potential to increase patient compliance. Since RA patients often struggle with frequent dosing regimes and severe symptoms, a substantial enhancement in therapeutic efficacy of MTX via the MSP (i.e., at the levels observed in CIA models reported here, i.e., about a 200% improvement in the reduction of RA score vs. conventional MTX) would represent a viable strategy to eliminate or reduce side effects by lowering the MTX dose while maintaining equal therapeutic outcome. In this way, RA patients could reduce long-term risks associated with high doses of MTX, such as hepatic and renal toxicity, while improving disease control and quality of life.

Unlike free MTX, which has a rapid release, the MSP system enables a slow and controlled release of the drug, maintaining therapeutic levels over extended periods. This could not only reduce injection frequency but also improve disease progression control.

Compared to other delivery systems, the MSP platform offers significant advantages in terms of scalability. Its production process is easily adaptable to large-scale manufacturing, ensuring consistent quality. Of course, the developmental path to industrialization and clinical deployment of the MSP is not immediate and would of course require further proofs and the implementation of a GMP production and several regulatory steps, including successful clinical trials.

Finally, we offer that the MSP could be applicable in other medical treatments beyond RA and beyond MTX, every time a slow delivery of an API is needed. In this respect, if confirmed by data in future studies, the MSP would represent a formidable asset to repurpose and reposition many existing drugs, providing a way to use them in a different manner just by introducing a slow-release mechanism, as seen for RA.

## 4. Conclusions

The results of this study convey the potential benefits of the proposed MSP platform for their use in the treatment of rheumatoid arthritis, laying the foundation for the subsequent development and optimization study needed to bring this approach to the clinical stage.

The formulation studies reported in this paper achieved homogeneous and stable batches of the MSP, capable of withstanding the freeze-drying process, while maintaining its characteristic size and drug loading release behaviors. The obtained powder is easily re-suspendable on demand at the time of SC administration. A sterile powder formulation is generally advantageous as it simplifies storage, reduces contamination risks, and allows for safe and easy injection, overcoming issues associated with liquid formulations, such as stability.

Our in vitro release studies showed that the MSP platform enables a much slower and sustained drug release compared to the drug alone, which ultimately could extend the therapeutic effect of a single injection at same given dose of MTX and reduce the frequency of administrations, largely benefiting patients both in terms of efficacy and quality of life. This was verified through preclinical efficacy studies, which showed that the MSP system clearly outperforms traditional subcutaneous MTX administration in terms of reduced severity of RA symptoms. The safety of the MSP was also investigated thoroughly, since safety is a prime prerequisite of any new formulation. In-depth toxicity assays were performed to confirm the safety profile of the MSP platform, both when tested alone and when loaded with the drug, finding that the MSP is well-tolerated with no significant side effects during preclinical testing. Furthermore, the verified absence of endotoxins in our batches ensures their compliance for parenteral administration, which is another crucial prerequisite for preventing adverse reactions in patients and guaranteeing the safety of any product for clinical use.

All these results as a whole position the MSP system as a promising solution to address current drug delivery challenges, with the potential to significantly improve patient treatment and clinical outcomes.

To expand on this work, foreseeable research directions would include, for example, (i) assessing long-term outcomes of RA treated by MTX-MSP, (ii) running a large dose-finding investigation to determine the optimal formulation of the MTX-MSP combination either for RA management or achieving precise clinical end-points of interest (e.g., a MSP formulation for either minimum size-effects, or for maximum effects, or best suited for a specific stage of RA, etc.), and (iii) elucidating the mechanisms underlying the observed outcomes of the MSP formulation compared to conventional MTX delivery. Among more fundamental questions related to RA, perhaps it is worth investigating the effect of estrogen on the therapeutic efficacy and the role of acidic environments on the kinetics of MTX release from the MSP, particularly in relation to MTX serum levels after SC injection.

Moreover, exploring alternative routes of administration beyond subcutaneous injection and other clinical needs beyond RA could greatly enhance the scope and applicability of this drug delivery platform to treat many more chronic and acute diseases. In principle, due to its compatibility with several drugs and biomolecules, the MSP technology might be considered in all those therapeutic settings where a DDP for slow release is beneficial, such as osteoarthritis, type 2 diabetes, cancer treatment, ophthalmic intravitreal injections, mesotherapy, etc. In addition, the chemo-physical nature of the MSP allows this DDP to interact with many drugs and biomolecules besides MTX, thus bringing an interesting opportunity to repurpose many existing drugs in the medical industry. In conclusion, the applicability of the MSP in the transition towards next generation precision and personalized medicine is potentially quite vast and diverse.

## Figures and Tables

**Table 1:** Histological scoring.

Histological Scoring			
Architectural Damage		Inflammatory Infiltrate	
No alterations	0	No inflammatory cells	0
Slight alterations	1	Rare isolated inflammatory cells	1
Moderate alterations	2	Focal infiltration of inflammatory cells	2
Severe alterations	3	Widespread infiltration of inflammatory cells	3

**Table 2:** Histological rheumatoid arthritis scoring.

Histological Rheumatoid Arthritis Scoring							
Structure		Cells		Matrix Staining		Tidemark Integrity	
Normal	0	Normal	0	Normal	0	Intact	1
Surface irregularities	1	Diffuse hypercelluarity	1	Slight reduction	1		
Pannus and surface irregularities	2	Clusters	2	Moderate reduction	2		
Clefts to transitional zone	3	Hypocellularity	3	Severe reduction	3	Destroyed	2
Clefts to calcified zone	4			No staining	4		
Complete disorganization	5						

**Table 3:** Particle size of all samples by SEM.

Formulation	Centrifuged	Lyophilized
	Dimensions (mm ± SD)	Dimensions (mm ± SD)
MSP	8.34 ± 0.35	7.44 ± 0.64
MTX-MSP	7.25 ± 0.55	6.87 ± 0.73

**Table 4:** Mean particle size distribution of unloaded and loaded MSP after lyophilization using Mastersizer^®^ 3000, when MSP are swollen. The ζ-potential analysis of all samples used a DLS. Endotoxin concentration in all samples after synthesis process. All analyses were performed in triplicate (n = 3) and results are expressed as the mean ± standard deviation.

Formulation	Mean Particle Size (μm) ± SD	ζ-Potential (mV) ± SD	Endotoxin (EU/mL)
MSP	34.5 ± 0.2	−36 ± 2	<0.01
MTX-MSP	60.8 ± 0.5	−43 ± 2	<0.01

## Data Availability

Data are available from the corresponding author upon reasonable request.

## Supplementary Materials

The following supporting information can be downloaded at: https://www.mdpi.com/article/10.3390/pharmaceutics16121593/s1, Figure S1. Confocal images of the MSP; Figure S2. Release profile changes over time for different formulations; Figure S3. Histopathological analysis for safety study; Figure S4. Heat map illustrating Architectural Damage and Inflammation Scores for each tested organ. Table S1. Effect of different concentration of the MSP, CL, and HA treatments in rats, complete blood count (CBC); Table S2. Rheumatoid Arthritis scores for the Right Knee; Table S3. Rheumatoid Arthritis scores for the left knee: Table S4. Rheumatoid Arthritis scores for the total knees: Table S5. Effect of different treatments of MTX, the MSP, and a combination of MTX-MSP treatment in rats: complete blood count (CBC).
