# Supplementary material for: Application of Microsponge Drug Platform to Enhance Methotrexate Administration in Rheumatoid Arthritis Therapy
Source: Pharmaceutics. 2024 Dec 13;16(12):1593. doi: 10.3390/pharmaceutics16121593 (PMC11676977; doi:10.3390/pharmaceutics16121593)
Supplement: Supplementary file 1 [file pharmaceutics-16-01593-s001.zip › pharmaceutics-3324889-supplementary.pdf]

Supplementary Materials

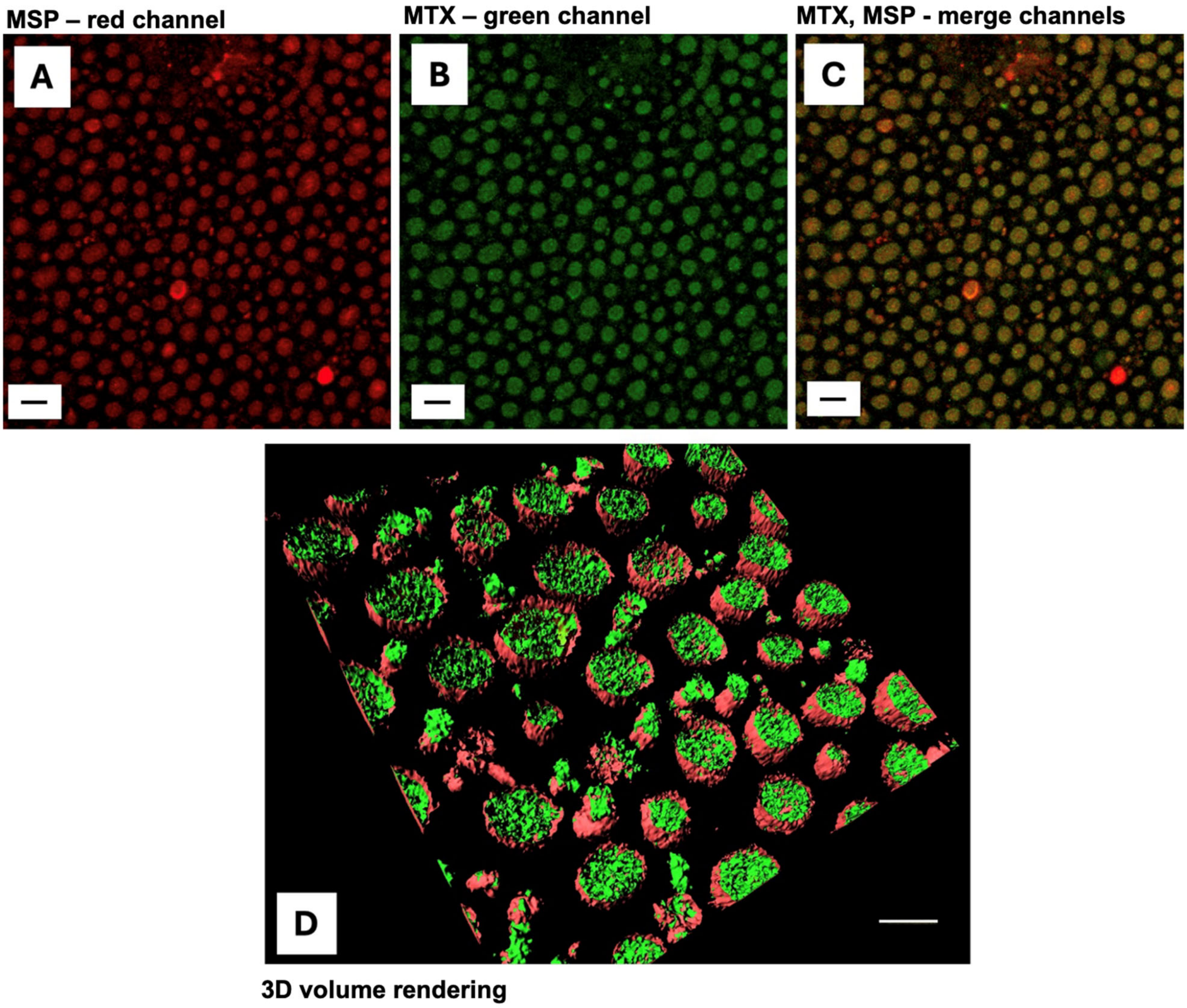

Figure S1. Confocal images of MSP. In red (A, MSP), green (B, MTX) and merge channels (C); magnification 60x, scale bar 10µm; Panel D: 3D rendering with isosurface confocal image acquired with 60x oil objective and 6x optical zoom (scale bar 4 µm) with a retractable surface (clipping plane) to visualize the MTX (green fluorescence) inside the MSP (red channel) (Imaris software, Bitplane).

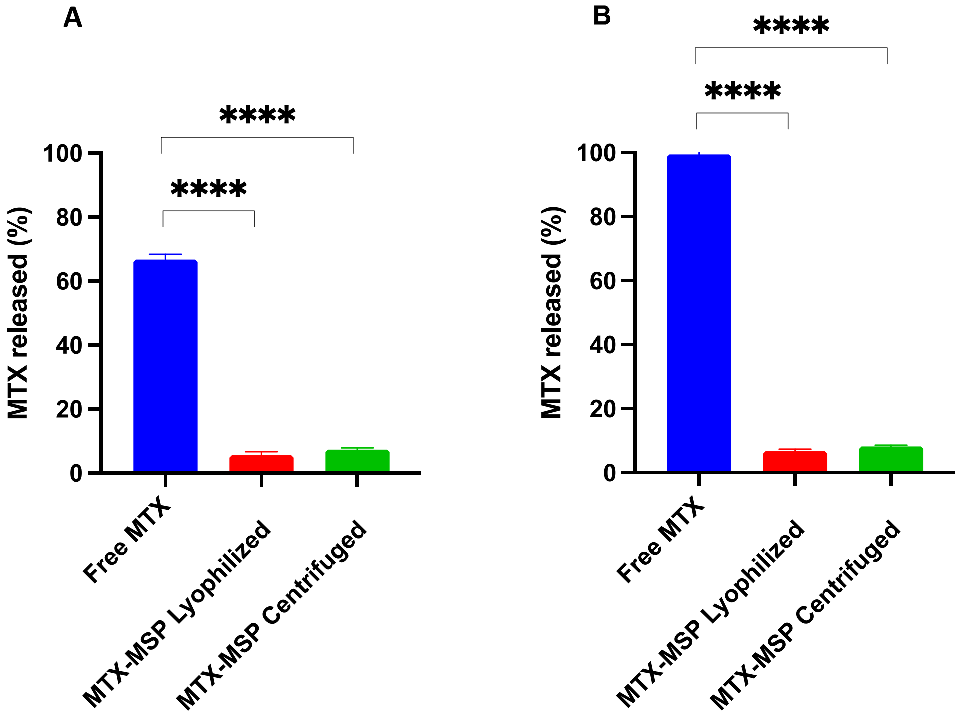

Figure S2. Release profile changes over time for different formulations. Panel A displays the changes in the release profile during the first hour for the different formulations. Panel B shows the corresponding changes observed during the second hour. The results are expressed as mean ± standard deviation and were performed in triplicate. ****p<0.0001 compared with Free MTX.

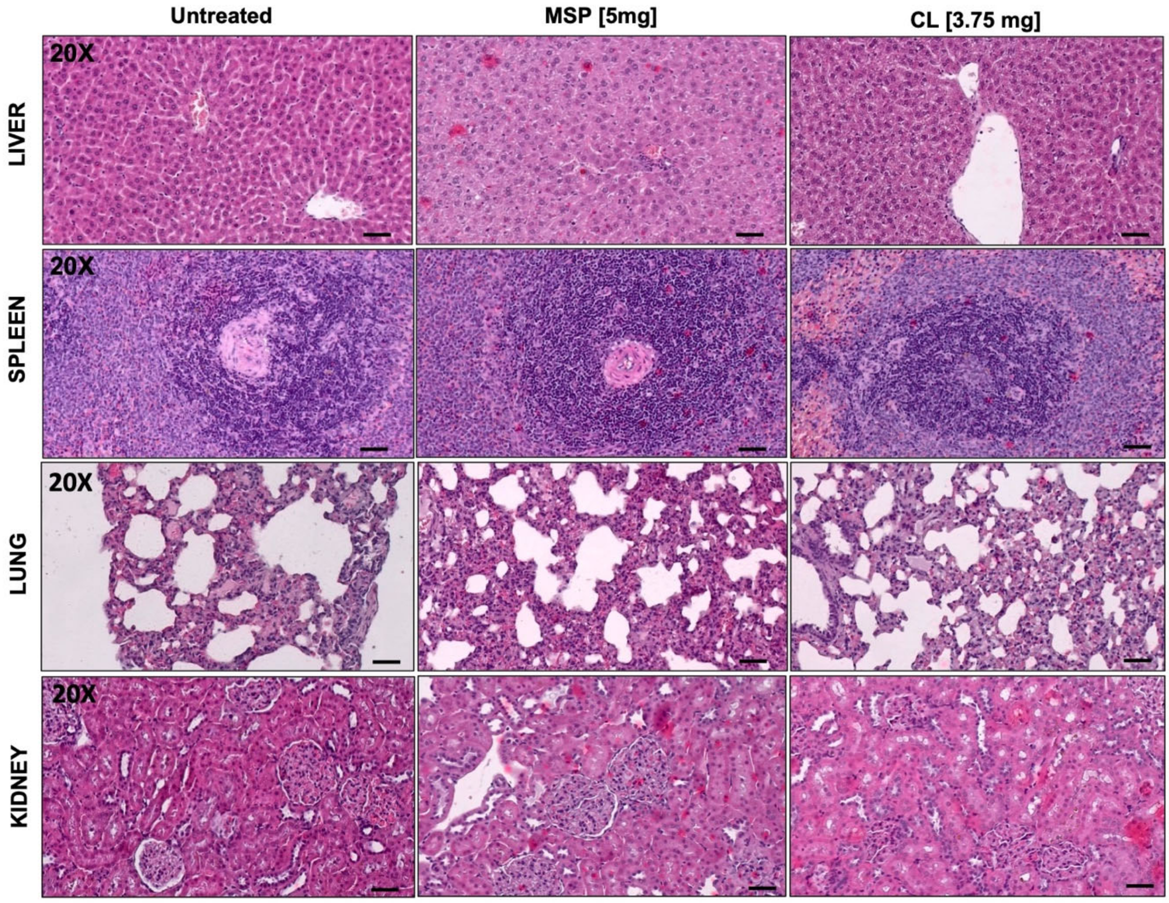

Figure S3. Histopathological analysis for safety study. Histological analysis of Knee Joint sections by H&E staining: (A) Liver; (B) Spleen; (C) Lung; (D) Kidney. 20 X magnification: Scale bar = 50 μm.

Table S1. Effect of different concentration of MSP, CL and HA treatments in rats: complete blood count (CBC). *=p<0.05; **=p<0.001 vs Negative Control group; #=p<0.05 vs Positive Control Group.

CBC	Reference values	Untreated Mean ± SD	MSP (1mg) Mean ± SD	MSP 5mg Mean ± SD	CL (0.75 mg) Mean ± SD	CL (3.75 mg) Mean ± SD	HA (0.25 mg) Mean ± SD
WBC (x10*9/L)	1.0-7.0	1.0±0.5	0.9±0.5	1.2±0.4	2.1 ± 1.6	1.1±0.2	1.1±0.4
LYM (%)	60.0-80.0	76 ± 9	75 ± 5	66 ± 6*	57 ± 7*	71 ± 3	62 ±16
MID (%)	4.0-12.0	5.1 ± 0.9	6 ± 1	8 ± 2#	10.6 ± 2.7*	7.0±1.4*	8.9±3.9*
GRAN (%)	15.0-35.0	19 ± 8	19 ± 4	27 ± 6*	32.6±4.8	22.2±1.7	29.5±11.9
LYM (x10*9/L)	0.6-5.4	0.8±0.4	0.9±0.4	0.7±0.2	1.1±0.7	0.8±0.2	0.6±0.1
MID (x10*9/L)	0.1-1.0	0.1±0.1	0.1±0.1	0.1±0.1	0.3±0.2	0.1±0.0	0.1±0.1
GRAN (x10*9/L)	0.3-2.8	0.2±0.1	0.2±0.1	0.3±0.1	0.7±0.7*	0.2±0.1*	0.4±0.2
RBC (x10*12/L)	6.0-9.0	6.9±0.3	6.8±0.3	6.1±0.6*	6.5±0.2*	6.7±0.2	6.5±0.3*
HGB (g/dL)	10.0-15.0	12.5±0.4	12.2±0.2	11.2±1.4#	11.5±0.2*	11.9±0.2*	11.5±0.4#
HCT (%)	35.0-55.0	42.2±1.8	42.4±2.4	37.4±4.3*	39.8±1.7*	40.6±2.3	39.8±1.9*
MCV (fL)	55.0-65.0	60.9±0.7	61.9±0.9	60.9±1.2	61.2±0.9	61.7±1.2	61.4±0.8
MCH (pg)	15.0-25.0	18.0±1.1	17.7±0.7	18.1±0.5	17.7±0.6	18.0±1.2	17.7±0.4
MCHC (g/dL)	25.0-35.0	29.6±1.7	28.7±1.4	29.9±0.6	29.9±1.2	29.3±1.8	28.9±0.7
PLT (x10*9/L)	200.0-800.0	230±52*	173±15	205±34	243±50	194±25	192±80
MPV (fL)	5.0-8.0	6.7±0.2	6.6±0.2	6.8±0.2	6.9±0.3	6.7±0.2	6.8±0.3
PDW (%)	0-99.0	6.6±0.2*	5.9±0.3	6.7±0.3	6.6±0.2	6.5±0.1	7.2±1.5
PCT (%)	0.01-99.00	0.15±0.04*	0.11±0.01	0.13±0.03	0.16±0.03	0.13±0.02	0.12±0.05
P-LCR (%)	0-99.0	9.4±1.3	9.3±1.8	9.6±2.3	11.0±2.6	9.4±2.4	9.2±2.8

Abbreviations: SD: Standard Deviation; WBC: White Blood Cell (WBC); LYM: Lymphocytes; MID: Mid-Range Cells; GRAN: Granulocytes; RBC: Red Blood Cell; HGB: Haemoglobin; HCT: Hematocrit; MCV: Mean Corpuscular Volume; MCH: Mean Corpuscular Haemoglobin; MCHC: Mean Corpuscular Hemoglobin Concentration; PLT: Platelet Count; MPV: Mean Platelet Volume; PDW: Platelet Distribution Width; PCT: Plateletcrit; P-LCR: Platelet-Large Cell Ratio.

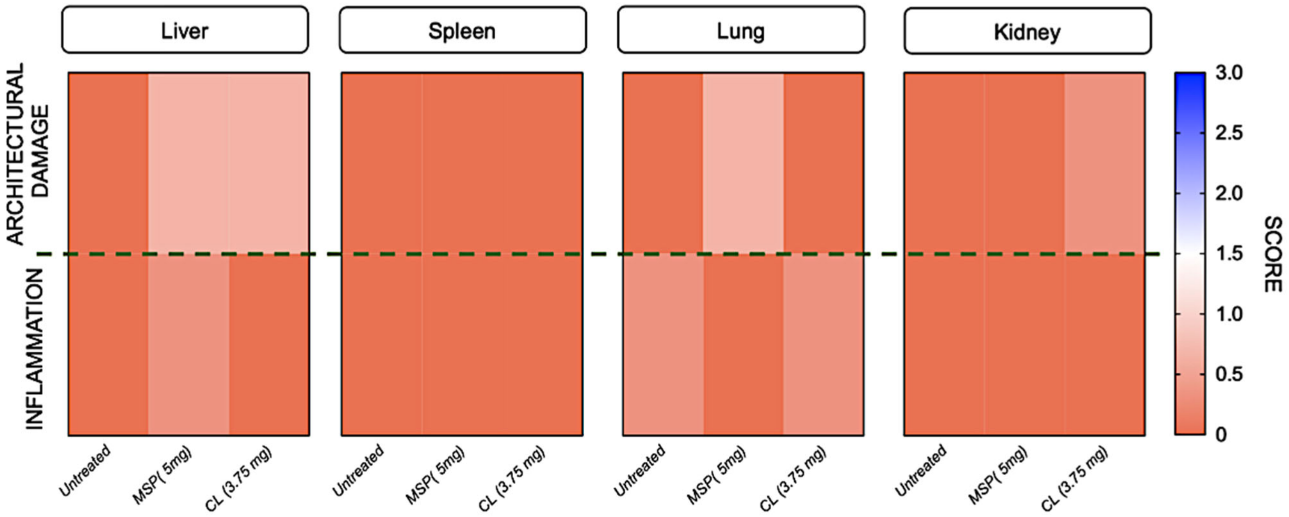

Figure S4. Heat map illustrating Architectural Damage and Inflammation Scores for each tested organ. In the heat map, orange indicates lower scores, while blue represents higher scores.

Table S2. Rheumatoid Arthritis scores for the Right Knee. The table also includes scores for the individual categories contributing to the overall RA score. SD: standard deviation.

**Right Knee**	Mean				Mean	
Groups	**Structure**	**Cells**	**Matrix**	**Tidemark**	**RA SCORE**	**SD**
**Ctrl -**	0.33	0	0.33	0	0.66	0.57
**Ctrl +**	2.25	2	1.50	1	6.75	1.50
**MSP**	1	0.50	1.25	0.50	3.25	2.21
**MTX**	0.66	0	0.33	0.66	1.66	1.15
**MTX-MSP**	0.33	0.33	1	1	2.66	0.57

Table S3. Rheumatoid Arthritis scores for the Left Knee. The table also includes scores for the individual categories contributing to the overall RA score. SD: standard deviation.

**Left Knee**	Mean				Mean	
Groups	**Structure**	**Cells**	**Matrix**	**Tidemark**	**RA SCORE**	**SD**
**Ctrl -**	0	0	0	0	0	0
**Ctrl +**	2	2	2.50	1	7.50	0.57
**MSP**	1.25	0.25	0.50	0.50	2.50	1.73
**MTX**	0	1	2	0.66	3.67	2.08
**MTX-MSP**	0.33	0	0	0.66	1	1.0

Table S4. Rheumatoid Arthritis scores for the Total Knees. The table also includes scores for the individual categories contributing to the overall RA score. SD: standard deviation.

**TOTAL KNEES**	Mean				Mean	
Groups	**Structure**	**Cells**	**Matrix**	**Tidemark**	**RA SCORE**	**SD**
**Ctrl -**	0.16	0	0.16	0	**0.33**	0.51
**Ctlr +**	2.12	2	2	1	**7.12**	1.12
**MSP**	1.12	0.37	0.87	0.50	**3.25**	1.98
**MTX**	0.33	0.50	1.16	0.66	**2.66**	1.86
**MTX-MSP**	0.33	0.16	0.50	0.83	**1.83**	1.16

Table S5. Effect of different treatments of MTX, MSP and a combination of MTX-MSP treatment in rats: complete blood count (CBC).
*=p<0.05 vs Negative Control Group
#=p<0.05 vs Positive Control Group

CBC	Reference values	Ctrl – Mean ± SD	Ctrl + Mean ± SD	MTX Mean ± SD	MSP Mean ± SD	MTX-MSP Mean ± SD
WBC (x10*9/L)	1.0-7.0	5.9±2.6	3.7±1.2	1.6±0.7#	4.9±1.6	6.0±2.3
LYMF (%)	60.0-80.0	77 ± 10	67 ±12	75 ± 5	71 ± 8	63.3 ± 0.9
MID (%)	4.0-12.0	6.3±1.5	7.7±4.8	5.3±1.1	6±1.7	9.1±0.4*
GRAN (%)	15.0-35.0	17.1±8.7	24.9±9.2	19.5±4.1	22.5±6.4	27.6±0.6
LYM (x10*9/L)	0.6-5.4	4.4±1.5	3.0±1.2	1.2±0.5*	3.4±0.9	3.8±1.4
MID (x10*9/L)	0.1-1.0	0.4±0.2	0.3±0.1	0.1±0.06	0.3±0.2#	0.5±0.1#
GRAN (x10*9/L)	0.3-2.8	1.1±0.9	0.8±0.1	0.3±0.1	1.1±0.5#	1.7±0.7
RBC (x10*12/L)	6.0-9.0	7.7±0.8	7.3±1.1	6.8±0.8	7.8±0.5	7.7±0.3
HGB (g/dL)	10.0-15.0	13.4±1.4	12.5±1.4	12.5±0.5	13.3±1.0	13.9±0.7
HCT (%)	35.0-55.0	47.8±6.3	44.1±7.2	42.3±4.5	48.2±2.9	48±3.1
MCV (fL)	55.0-65.0	62.3±1.9	60.5±1.6	61.4±0.9	61.9±1.0	62.1±1.3
MCH (pg)	15.0-25.0	17.4±0.1	17.2±1.1	17.1±0.2	17±0.7	18±0.3*
MCHC (g/dL)	25.0-35.0	28.1±0.8	28.6±2.6	28.6±0.5	27.6±1.1	29±0.6
PLT (x10*9/L)	200.0-800.0	201±84	195±71	199±70	241±4	212±50
MPV (fL)	5.0-8.0	7.4±0.5	6.8±0.7	7.2±0.06	6.8±0.5	6.9±0.4
PDW (%)	0-99.0	8.3±1.7	7.8±1.6	7.2±0.91	7.2±0.5	7.7±0.5
PCT (%)	0.01-99.00	0.1±0.05	0.1±0.06	0.1±0.09	0.2±0.01	0.1±0.04
P-LCR (%)	0-99.0	15.4±4.1	9.3±6.0	13.1±3.0	9.5±4.7	9.5±5.8

Abbreviations: SD: Standard Deviation; WBC: White Blood Cell (WBC); LYM: Lymphocytes; MID: Mid-Range Cells; GRAN: Granulocytes; RBC: Red Blood Cell; HGB: Haemoglobin; HCT: Hematocrit; MCV: Mean Corpuscular Volume; MCH: Mean Corpuscular Haemoglobin; MCHC: Mean Corpuscular Hemoglobin Concentration; PLT: Platelet Count; MPV: Mean Platelet Volume; PDW: Platelet Distribution Width; PCT: Plateletcrit; P-LCR: Platelet-Large Cell Ratio.
